# Assessing the impact of neurosurgery and neuroanatomy simulation using 3D non-cadaveric models amongst selected African medical students

**DOI:** 10.3389/fmedt.2023.1190096

**Published:** 2023-05-04

**Authors:** Berjo Dongmo Takoutsing, Ubraine Njineck Wunde, Yvan Zolo, Geneviève Endalle, Dieunedort Arnold Menga Djaowé, Léopoldine Sybile Ngoune Tatsadjieu, Ines Miste Zourmba, Aminatou Dadda, Roland Ndouh Nchufor, Cyrille Duquesne Nkouonlack, Ernestine Renée Atangana Bikono, Jean Paul Oumarou Magadji, Casimir Fankem, Alain Bertrand Tchoupo Jibia, Ignatius Esene

**Affiliations:** ^1^ Research Division, Winners Foundation, Yaounde, Cameroon; ^2^ Research Department, Association of Future African Neurosurgeons, Yaounde, Cameroon; ^3^ Faculty of Health Sciences, University of Bamenda, Bambili, Cameroon; ^4^ Global Surgery Division, Faculty of Health Science, University of Cape Town, Cape Town, South Africa; ^5^ Faculty of Health Sciences, University of Buea, Buea, Cameroon; ^6^ Faculty of Medicine and Biomedical Sciences, University of Garoua, Garoua, Cameroon; ^7^ Faculty of Medicine and Biomedical Sciences, University of Yaounde I, Yaounde, Cameroon; ^8^ Yaounde Military Hospital, Yaounde, Cameroon; ^9^ Yaounde Emergency Center, Yaounde, Cameroon

**Keywords:** brain, cadaver, Cameroon, neuroanatomy, neurosurgery, simulator, students, virtual reality

## Abstract

**Background:**

Laboratory dissections are essential to acquire practical skills to perform neurosurgical procedures. Despite being traditionally done on cadavers, they are often unavailable and suffer from cultural barriers in the African context. Non-cadaveric UpSurgeOn neurosurgery models have been developed to bridge this barrier, providing an almost similar experience with the human body. This study aimed to assess the impact of the UpSurgeOn hands-on-touch non-cadaver model training amongst selected Cameroon medical students.

**Methods:**

An anonymous 35-item questionnaire was distributed online using Google drive systems to medical students who attended UpSurgeOn's hands-on-touch non-cadaver model training course. These questions aimed to capture data on previous experience with neuroanatomy and neurosurgery practicals and the perception, attitudes, and impact of the UpSurgeOn neurosurgery tool.

**Results:**

Eighty-six students completed the survey. The mean age was 21.2 ± 1.868 years, 61.6% were males with 62.8% of respondents being medical students in preclinical years. Before the training, 29.4% had a fair knowledge of neuroanatomy. Textbooks and Youtube videos were the main sources of neuroanatomy and neurosurgery knowledge for more than half of the respondents. Up to 91.5% had no prior exposure to a neuroanatomy/neurosurgery cadaver laboratory dissection, and 22.6% and 17.6% had witnessed and performed at least one craniotomy before, respectively. There were 11.1%, 15.5%, and 31.3% of our respondents who had used a surgical microscope, a neurosurgical instrument, and the UpSurgeOn Neurosurgery tool before, respectively. The majority perceived the UpSurgeOn tool easy to use and felt they needed to learn just a few things before getting going with the box. Most thought of increasing the use of the UpSurgeOn Box and saw the need to be part of the training curriculum. Finally, the majority felt this tool helped to increase familiarity and acquire neurosurgical skills, and to develop the orientation skills needed during neurosurgical approaches.

**Conclusion:**

Undergraduate exposure to traditional neurosurgery/neuroanatomy labs is limited in Cameroon. Neurosurgery/neuroanatomy practical skills are gained essentially using non-practical means. Most students found the UpSurgeOn tool user-friendly, saw the need to incorporate it as part of their training, and perceived it to be essential in getting acquainted with neurosurgical skills.

## Introduction

Neurosurgery is a fast-growing medical specialty in Africa (1, 2). Africa is disproportionately affected by the high burden of neurosurgical conditions (3), and the vast majority of African medical students have limited exposure to neurosurgery (4). Neurosurgery being fundamentally a surgical discipline requires extensive knowledge of neuroanatomy (5). A recent survey among aspiring African neurosurgeons showed that the vast majority of students have little or no exposure to neurosurgical procedures (4). This highlights the scarcity of avenues for students to gain exposure and nurture their interest in the field. Published literature has shown that an essential long-term strategy in reducing this workforce deficit would include providing medical students with educational and career development opportunities related to neurosurgery (6). Traditional cadaver dissection has been viewed as the best way to study and understand the anatomy of the human body (7). However, some drawbacks and limitations must be taken into account. Neurosurgical anatomy is highly specific to the human brain and does not compare well with animal specimens. Moreover, the management of cadaveric models entails ethical concerns and high maintenance costs, as well as specific and highly equipped structures for their preservation (2). Moreover, many anatomists believe a medical student-cadaver relationship is essential in developing surgical skills and in developing proper attitudes toward patients (8, 9). Unfortunately, in sub-Saharan Africa, neurosurgical laboratories are limited, and the few available are faced with cultural, ethical, infrastructure, and funding barriers (10, 11). Recently, new techniques and computer-based programs have changed the vision and approach to teaching human anatomy courses (12). Simulation training using 3D printed models such as the UpSurgeOn non-cadaveric models (UpSurgeOn SRL, Via Cascina Venina, 7/U, Assago, Milan, Italy) is rapidly gaining popularity in high-income countries (13). UpSurgeOn is a rising enterprise with the mission to improve psychomotor performance in microsurgeries through the application of tearing virtual and physical modeling and simulation. The UpSurgeOn Academy is a technologically sophisticated collection of both virtual and physical innovations meant to promote mental and manual training while also supporting psychomotor training, and advancing cognitive and motor skills. Its mission is to fill the gap between theory and practice in neurosurgery (14). These models allow students to improve their technical surgical skills and knowledge at every stage of their education and ease their learning of neurosurgery and neuroanatomy (15). In doing so, technical skills are not only greatly improved, but the repetitive contact with the surgical field is intended to increase the number of well-prepared candidates for neurosurgical residency positions (4). In the African context, the utilization of virtual reality is not common due to the rudimentary nature of telecommunication technologies (16). Using 3D-printed models in place of human specimens could be better suited for this context where even an electric power supply is not promised. Studies have shown a positive perception, attitudes, and improvement of technical skills amongst medical students following hands-on-touch non-cadaver model training in surgery-related domains. However, to our knowledge, no study has evaluated the perception and attitudes of medical students in Cameroon as well as the improvement of their psychomotor skills following a hands-on-touch non-cadaver neuroanatomy and neurosurgery training models.

## Study aims

This study aimed to evaluate the perception, attitudes, and impact of the UpSurgeOn hands-on-touch non-cadaver training model amongst medical students in Cameroon.

## Methods

### Course design

**Figure 1:**
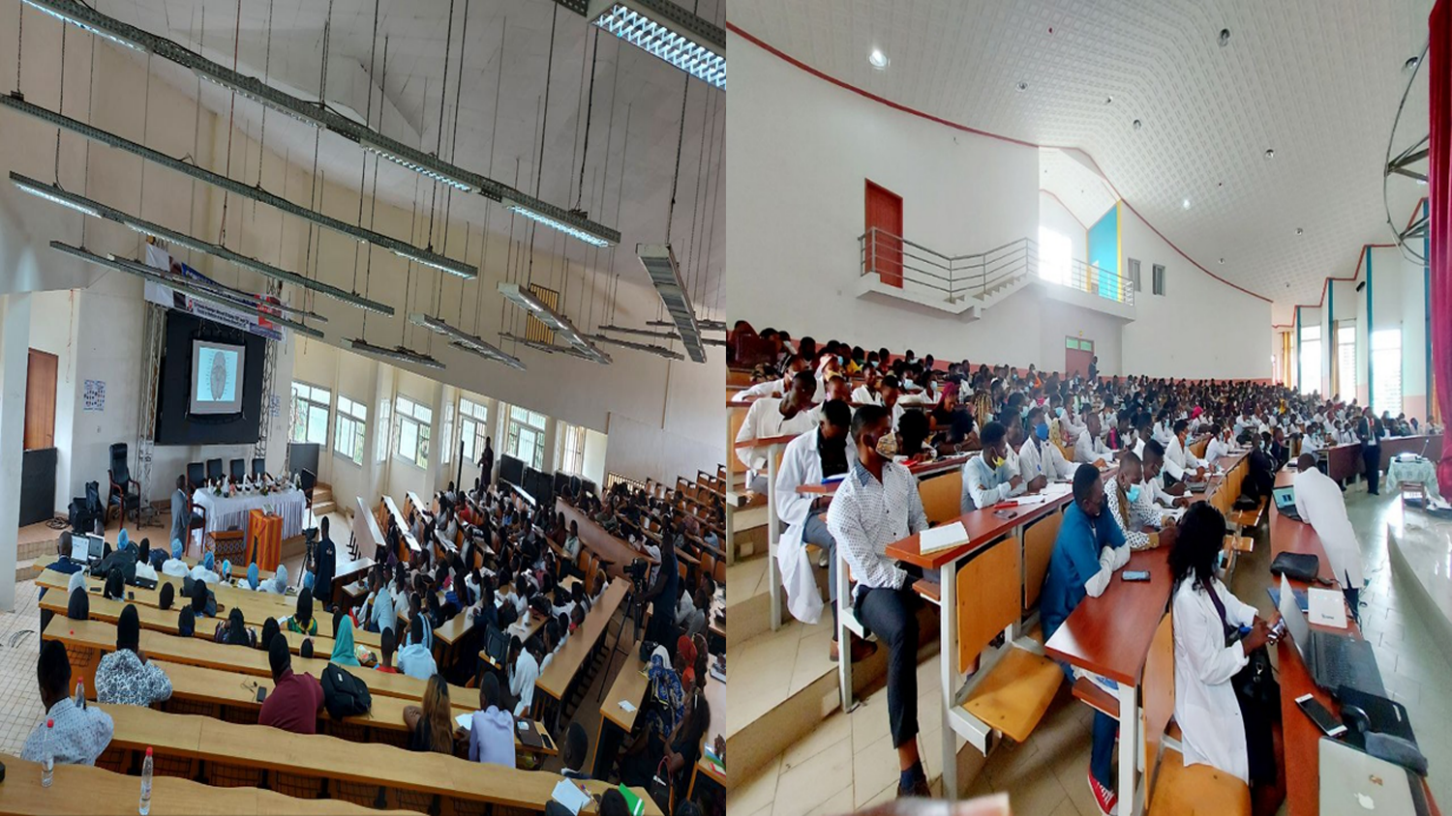
First phase of the training. Medical students receiving lectures on basic neurosciences delivered by a consultant neurosurgeon.

**Figure 2:**
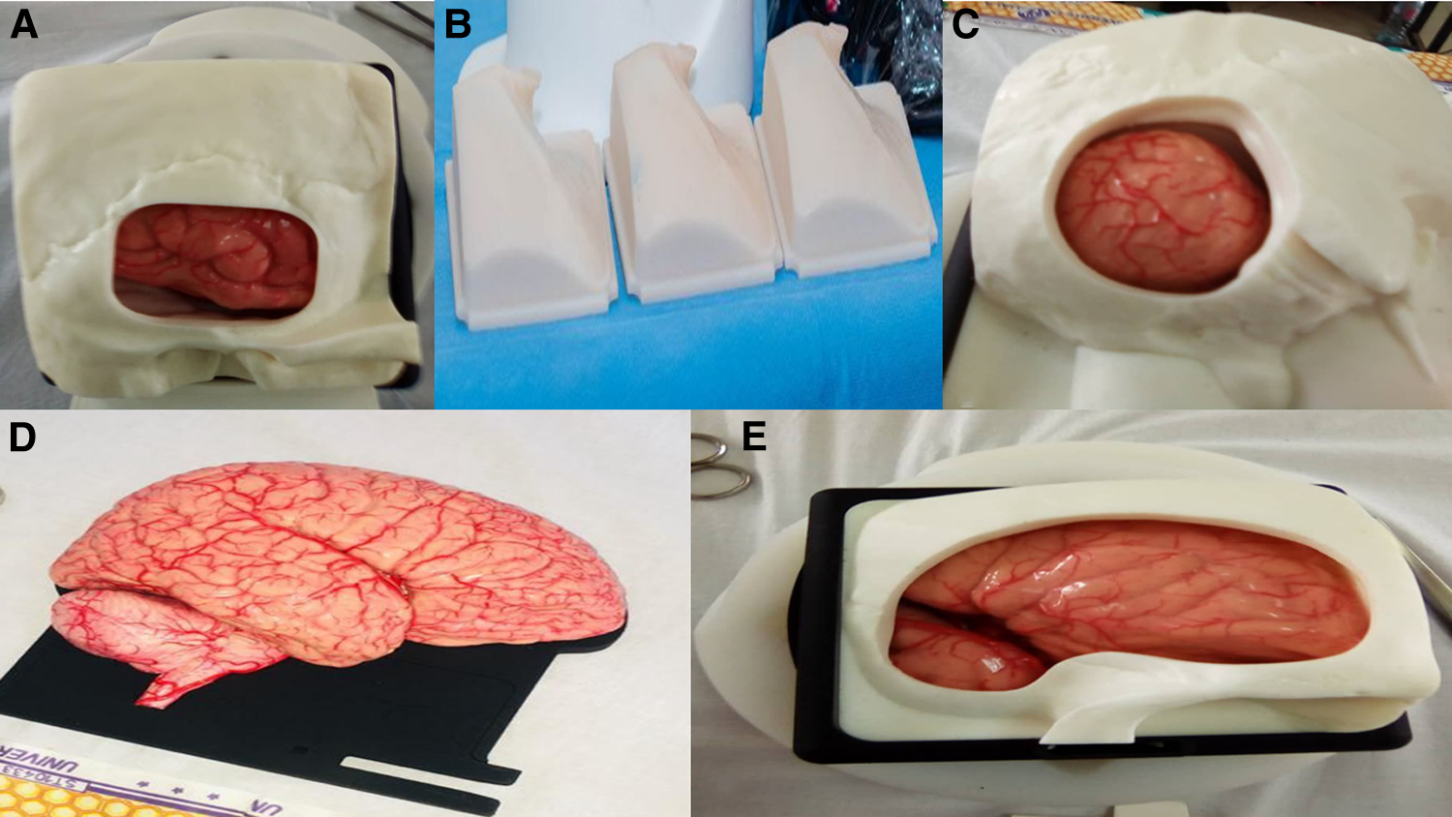
(**A–E**) UpSurgeOn non-cadaveric neuroanatomy models. (**A**) Temporal approach simulator model. (**B**) Disposable skulls. (**C**) Retrosigmoid approach simulator box. (**D**) Life-size human encephalon model. (**E**) Simulation box for brain aneurysm clipping.

**Figure 3:**
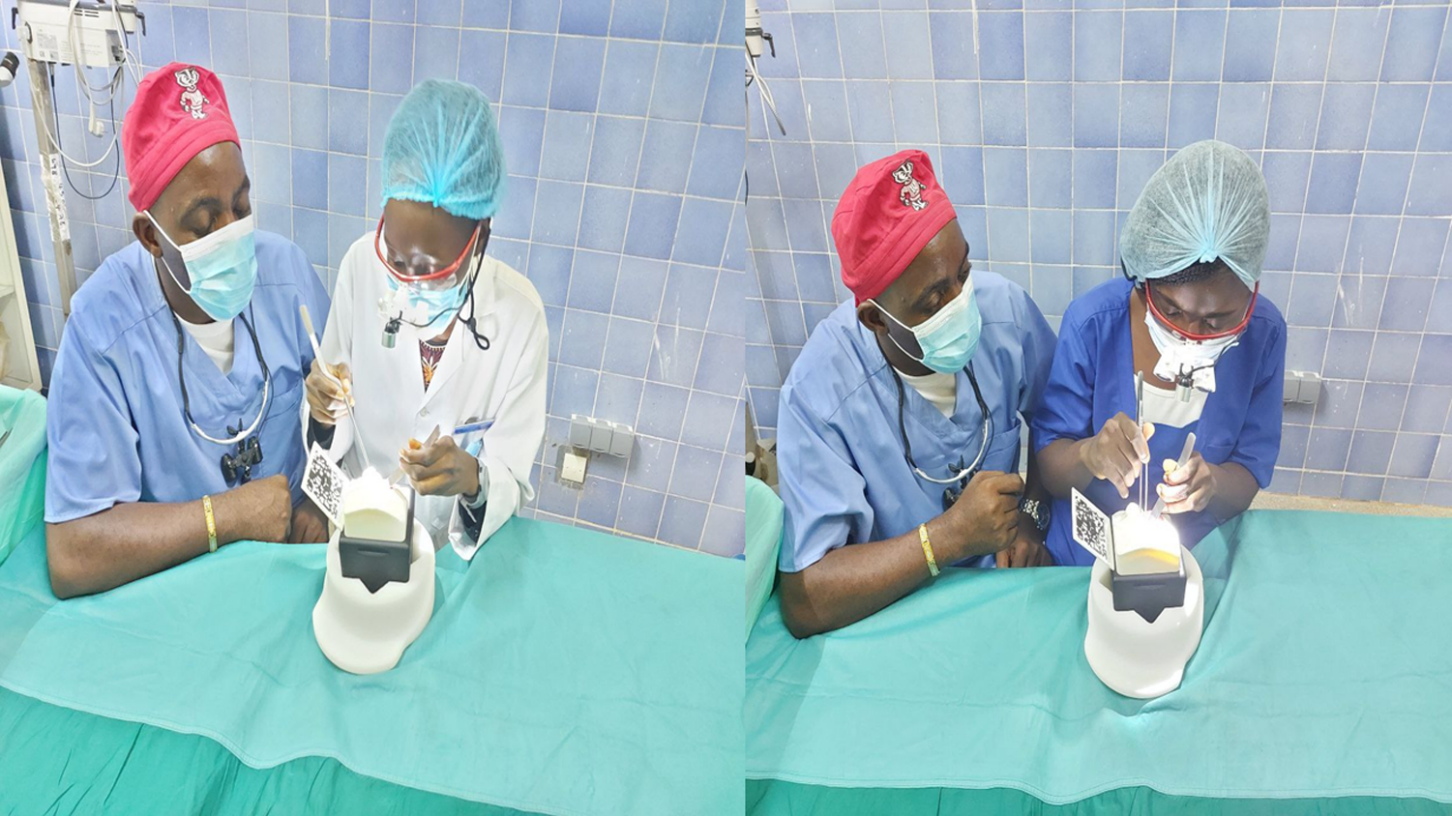
Second phase of the training. Medical students having a hands-on appraisal and exploring the brain structures of the simulation boxes with the help of a microscope under the guidance of a consultant neurosurgeon. All individuals in the photo gave their consent for publication in this article.

The UpSurgeOn hands-on-touch non-cadaver training was biphasic and took place in four medical schools in Cameroon; the universities of Bamenda, Buea, Garoua, Douala, and the Yaounde military hospital. The first phase consisted of lectures on neuroembryology, neuroanatomy, and neurophysiology presented by consultant neurosurgeons, senior residents, and members of the Association of Future African Neurosurgeons (Figure 1). The second phase was practical and consisted of hands-on-touch learning using the UpSurgeOn non-cadaveric neuroanatomy models (Figure 2). Four to five students or neurosurgery residents operated on the same non-cadaver brain box models under the supervision of at least one neurosurgeon tutor (Figure 3).

**Figure 4:**
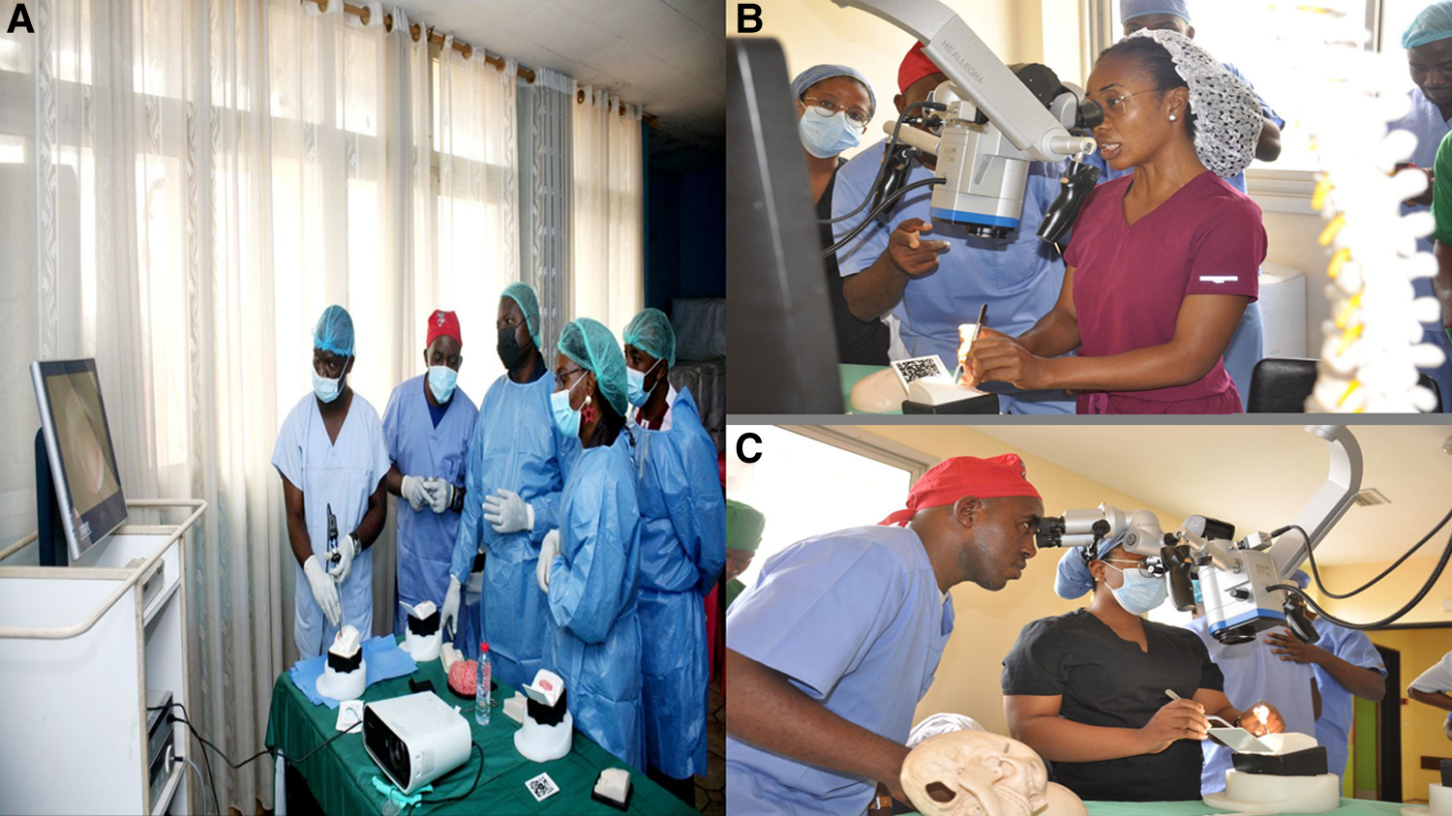
(**A–C**) Practical session using the surgical microscope, endoscope and simulator. (**A**) Residents and Neurosurgeons demonstrating the structures of the brain as seen under an endoscope. (**B,C**) Medical students performing a surgical exploration of brain organs with the help of a binocular microscope under the guidance of a neurosurgeon. All individuals in the photo gave their consent for publication in this article.

The overall number of participants in attendance was over 1,000. All participants in the training were aspiring neurosurgeons (medical students, early career physicians, or neurosurgery residents) with basic knowledge of embryology, anatomy, and physiology of the central nervous system and the skull. The course was run for eight hours on a single day at the various institutions mentioned above. All necessary surgical instruments to perform the surgery were provided, and the procedures mimicked the real surgical setting (Figure 4). The procedures included craniectomies, skull drilling, exploration of intracranial aneurysms, and skull base visualization just to name a few.

### Data collection tool

We designed a standardized anonymous questionnaire using Google Forms (Google, USA) in Cameroon's official languages (French and English). The questionnaire was validated by IE (consultant and academic neurosurgeon). A convenient sampling method was used to recruit our study of participants from the students who attended the course. This 35-item questionnaire featured a combination of multiple-choice and Likert-scale questions divided into eight sections. These questions aimed to capture data on (i) basic sociodemographics; (ii) previous experience and exposure to neuroanatomy; (iii) previous experience and exposure to neurosurgery; (iv) previous experience with the UpSurgeOn neurosurgery tool; (v) perception of the UpSurgeOn neurosurgery tool; (vi) attitudes towards the UpSurgeOn neurosurgery tool; (vii) impact of the UpSurgeOn neurosurgery tool to neurosurgical training (Supplementary Material).

### Data collection

The questionnaire was distributed following the training via the Whatsapp and Telegram social media platforms of the participants. The anonymized dataset collected is available on the Open Science Framework (https://osf.io/dyfh5/).

### Statistical analysis

The data collected were tidied on Excel 2016 (Microsoft Corp., USA). Summary descriptive statistics were generated, and the Chi-Square test was used in SPSS v26 (IBM, USA). To ease the analysis, the Likert scale was broken down into three categories. Strongly agree and agree merged as agree, and strongly disagree and disagree merged as disagree. The threshold of significance was set at *p* < 0.05.

## Results

### Demographics

Cameroon counts nine medical schools with a population of about 28.4 milion people and a density of 56/km2 (17). Its Surgical Preparedness Index is 81 +/− 12.5 which is one of the highest in central Africa, indicating that Cameroon has a better healthcare system than most other LMICs (18). We recruited a total of 86 students from six medical schools in Cameroon who attended the UpSurgeOn training and completed the post-training questionnaires. Amongst the respondents, 61.6% were male (*n* = 53) and the mean age was 21.2 ± 1.868 years. Up to 62.8% (*n* = 54) of respondents were in their preclinical years of medical school, while 37.2% (*n* = 32) were in their clinical years of medical school.

### Previous experience and exposure to neuroanatomy

**Figure 5:**
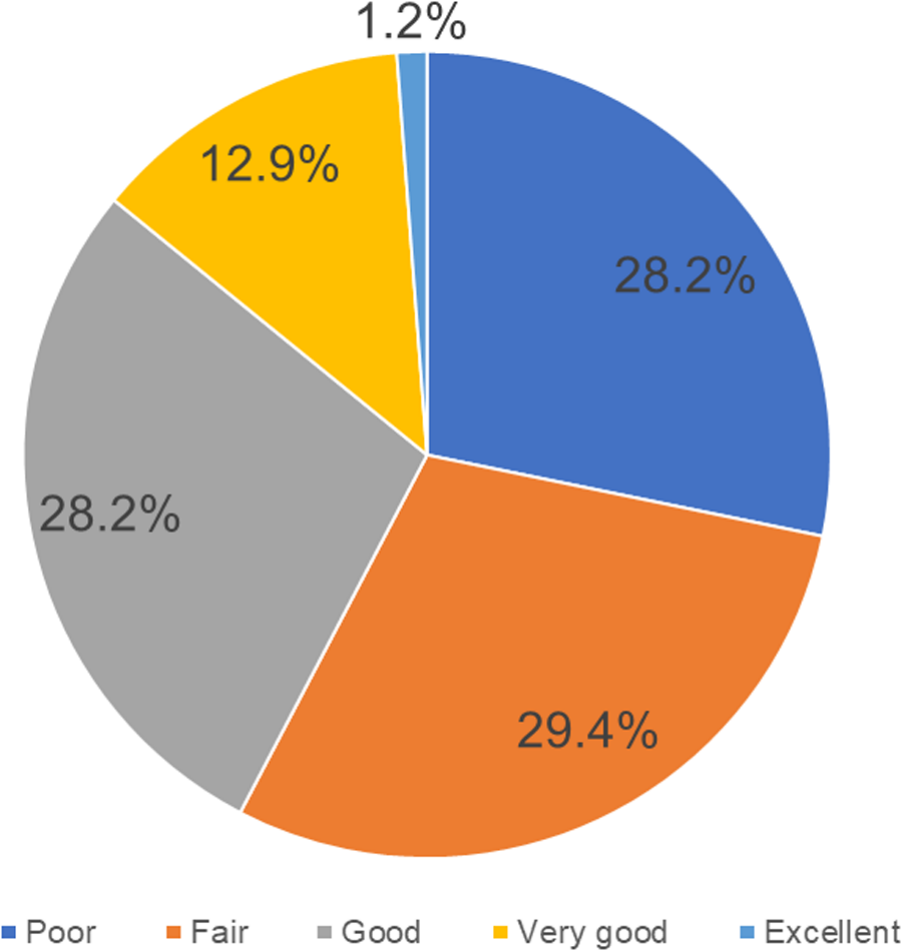
Neuroanatomy knowledge prior to the UpSurgeOn course (*N* = 85).

**Figure 6:**
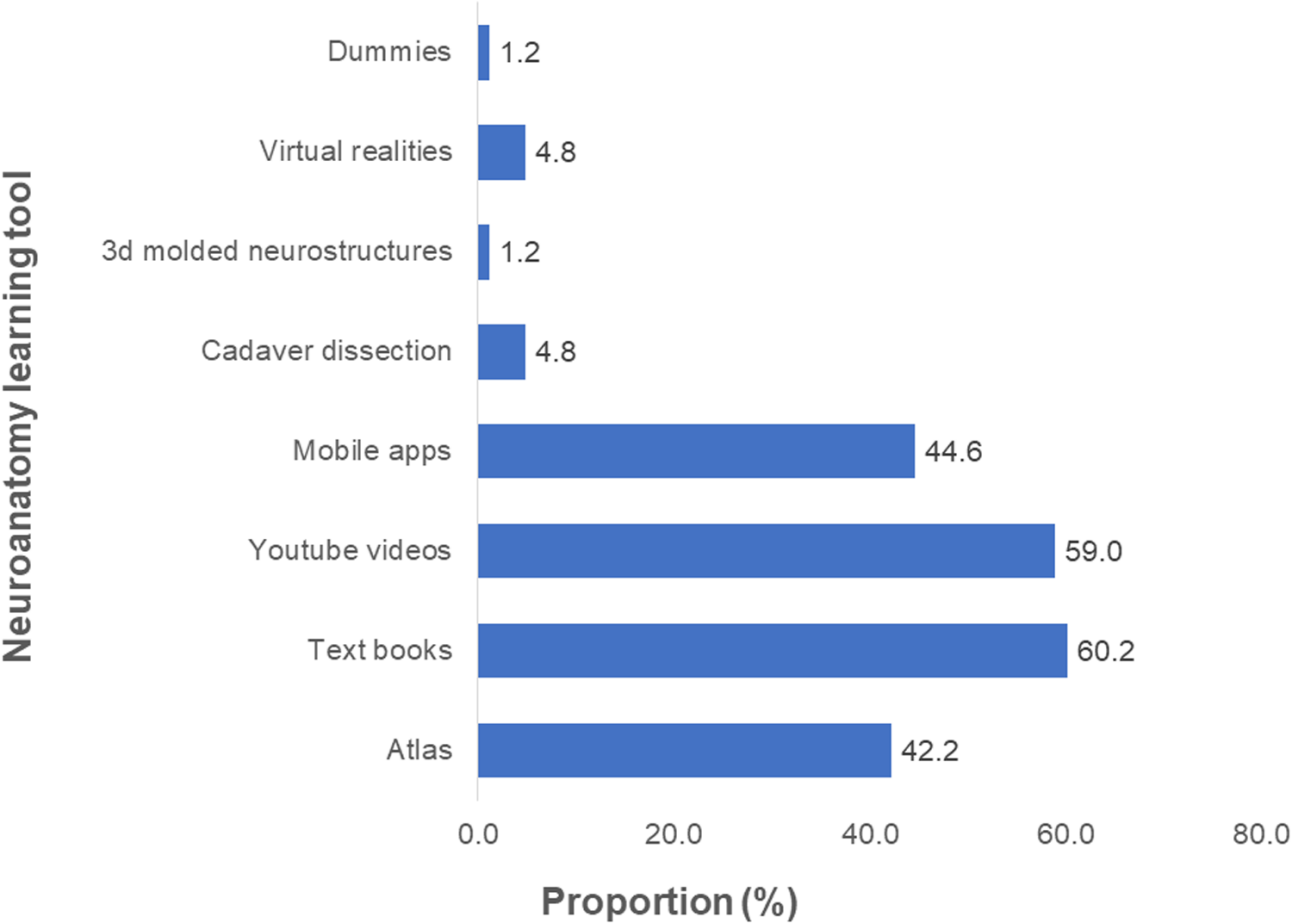
Proportions of different learning tools used to acquire knowledge in neuroanatomy prior to the UpSurgeOn course (*N* = 83).

Before the training, most participants had a fair knowledge of neuroanatomy (*n* = 25, 29.4%) (Figure 5). Textbooks (*n* = 50, 60.2%) and Youtube videos (*n* = 49, 59.0%) were the most common neuroanatomy learning tool employed (Figure 6), and 91.5% (*n* = 75) had never performed a neuroanatomy/neurosurgery cadaver lab dissection.

### Previous experience and exposure to neurosurgery

**Figure 7:**
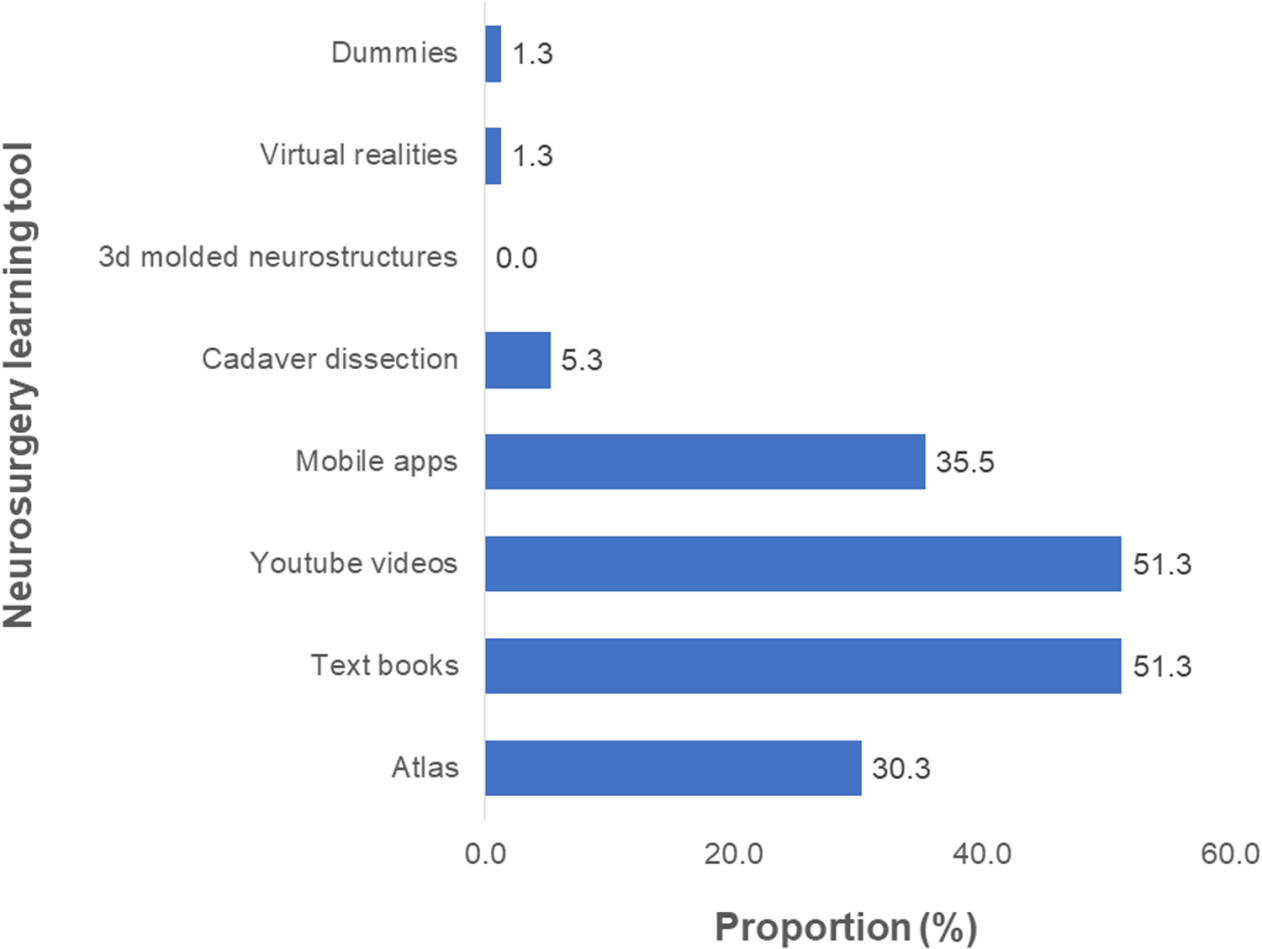
Proportions of different learning tools used to acquire knowledge in neurosurgery prior to the UpSurgeOn course (*N* = 76).

**Figure 8:**
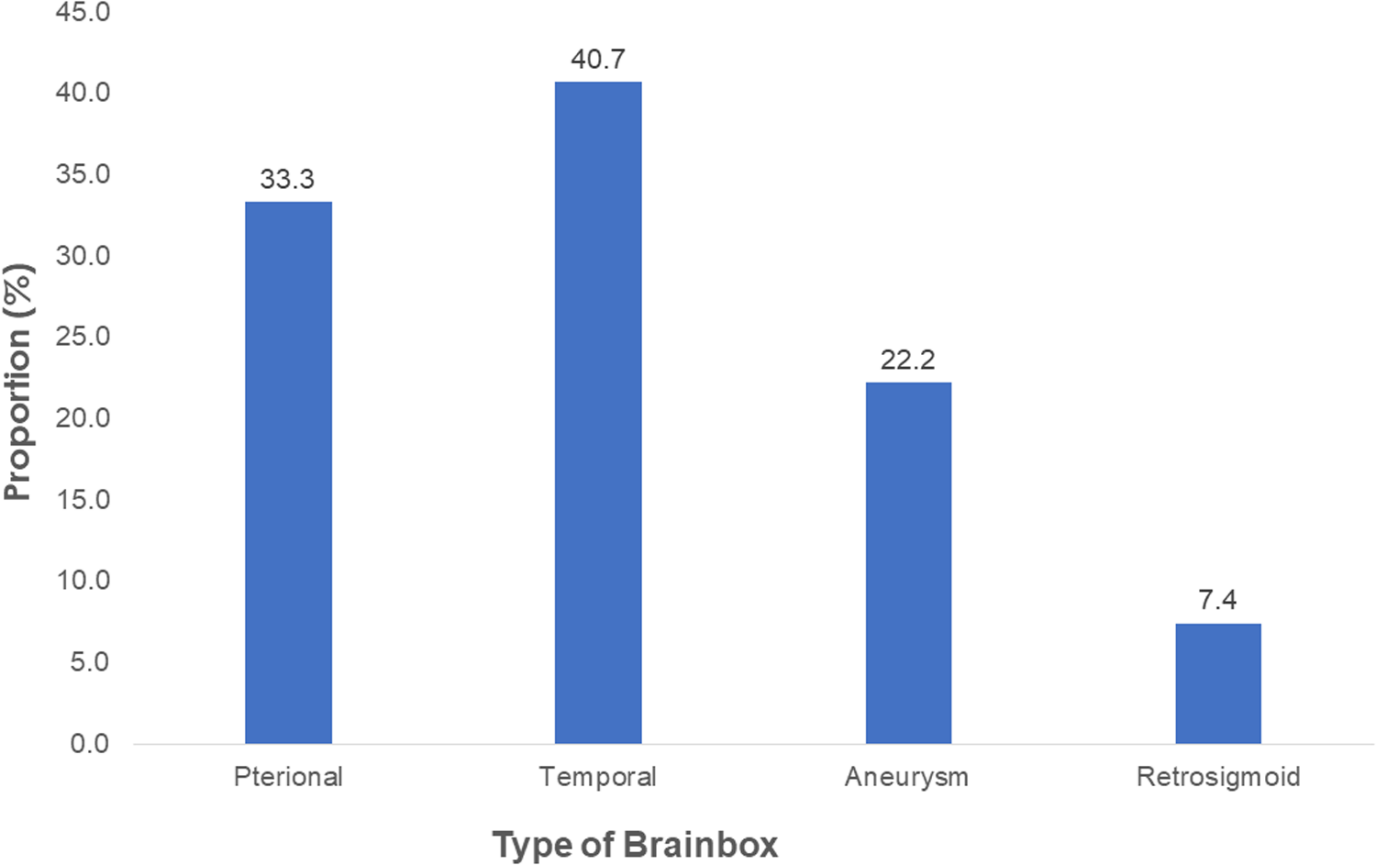
Frequencies of craniotomy brainboxes to which participants who performed or witnessed a craniotomy were exposed prior to the UpSurgeOn course (*N* = 27).

For neurosurgery experience, Textbooks, and Youtube videos were the most common (*n* = 39, 51.3%) neurosurgery learning tools used before the training (Figure 7). Only 22.6% (*n* = 19, *N* = 84) and 17.6% (*n* = 15, *N* = 85) had witnessed and performed at least one craniotomy, respectively. Of those with prior craniotomy experience, 17.9% (*n* = 5) were main operators, 25% (*n* = 7) were second operators, and the temporal box was the most common brain box (*n* = 11, 40.7%) to which they were exposed (Figure 8). Previous surgical microscopes and neurosurgical instrument use were common in 11.1% (*n* = 9) and 15.5% (*n* = 13) of the participants, respectively.

### Perception of the UpSurgeOn neurosurgery tool

The majority found UpSurgeOn Box easy to use (60.9%), needed to learn just a few things before getting acquainted with the UpSurgeOn Box (60%), and neurovascular structures and skull base anatomy were realistic and appropriately detailed for surgical orientation (58.6%) (Table 1).

**Table 1:** Student perception of the UpSurgeOn neurosurgery tool.

Perception	Disagree [*n* (%)]	Neutral [*n* (%)]	Agree [*n* (%)]
1. The BrainBox had an anatomically accurate surface of brain/cerebellum	4 (5.8)	26 (37.7)	29 (56.5)
2. Neurovascular structures and skull base anatomy were realistic and appropriately detailed for surgical orientation	7 (10.0)	22 (31.4)	41 (58.6)
3. The tactile feedback to manipulation of the brain/cerebellum was realistic	3 (4.3)	26 (37.7)	40 (58.0)
4. Feedbacks in the use of a high-speed drill and dissection instruments were realistic	4 (5.8)	33 (47.8)	32 (46.4)
5. The drilling experience was similar to the real skull	4 (5.7)	31 (44.3)	35 (50.0)
6. Dural opening and suturing was realistic	5 (7.2)	37 (53.6)	27 (39.1)
7. I found UpSurgeOn Box easy to use	5 (7.2)	22 (31.9)	42 (60.9)
8. I found the various functions in UpSurgeOn Box were well integrated	5 (7.5)	24 (35.8)	38 (56.7)
9. I thought there was much consistency in UpSurgeOn Box	5 (7.6)	28 (42.4)	33 (50.0)
10. I felt very confident using Upsurgeon Box	4 (6.2)	25 (38.5)	36 (55.4)
11. I needed to learn a few things before I could get going with UpSurgeOn Box	6 (9.2)	20 (30.8)	39 (60.0)

### Attitudes towards the UpSurgeOn neurosurgery tool

Most would like to use the UpSurgeOn Box frequently (76.7%) and advocate for the incorporation of this method of training into the standard training curriculum (67.1%) (Table 2). However, only 24.6% agreed that they would not need the support of a technical person to be able to use UpSurgeOn Box (Table 2).

**Table 2:** Student's attitude towards the UpSurgeOn neurosurgery tool.

Attitudes	Disagree [*n* (%)]	Neutral [*n* (%)]	Agree [*n* (%)]
1. This method of training should be part of a standard training curriculum, in addition to traditional training methods	7 (10.0)	16 (22.9)	47 (67.1)
2. I think that I would like to use UpSurgeOn Box frequently	4 (5.5)	13 (17.8)	56 (76.7)
3. I think that I would NOT need the support of a technical person to be able to use UpSurgeOn Box	25 (36.2)	27 (39.1)	17 (24.6)
4. I would imagine that most people would learn to use UpSurgeOn Box very quickly	4 (30.9)	21 (5.9)	43 (63.2)

### Impact of the UpSurgeOn neurosurgery tool

**Up to** 68.7% (*n* = 57) had no use of the UpSurgeOn Neurosurgery App and the AR simulator. More than half of the participants affirm that using this model has helped to increase their familiarity and to acquire neurosurgical skills (62.3%), and has helped to develop their orientation skills needed during neurosurgical approach, in addition to traditional resources (59.7%) (Table 3).

**Table 3:** Impact of the UpSurgeOn neurosurgery tool on students.

Impact	Disagree [*n* (%)]	Neutral [*n* (%)]	Agree [*n* (%)]
1. The Neurosurgery App and the AR simulator help to develop the orientation skills needed during neurosurgical approach, in addition to traditional resources.	3 (4.2)	26 (36.1)	43 (59.7)
2. Using this model helps to increase familiarity and to acquire neurosurgical skills	4 (5.8)	22 (31.9)	43 (62.3)
3. After using this model I feel more confident in the use of neurosurgical instruments	10 (14.5)	28 (40.6)	31 (44.9)
4. After using this model I feel more confident in the use of the surgical microscope	8 (12.1)	20 (45.1)	38 (42.4)
5. The sequence of mental training (app), hybrid training (Augmented Reality) and manual training (BrainBox) is an effective method of training in order to fill the gap between theoretical knowledge and practice on a real patient/cadaver	7 (10.4)	25 (37.3)	35 (52.2)

## Discussion

### Key findings

To the best of our knowledge, this study is the first to assess access the perception, impact, and attitudes towards using 3D printed non-cadaveric models for neurosurgery/neuroanatomy education in Africa. Our study highlights that knowledge of neuroanatomy wasn’t optimal, traditional sources of learning were used and exposure to neuroanatomy/neurosurgery cadaver lab dissection was scarce in this context. Also, there was little exposure to neurosurgical procedures and experience with the use of a surgical microscope/neurosurgical equipment. Moreover, the majority were using the UpSurgeOn app for the first time, found the box easy to use, were willing to use it more frequently, were happy for it to be integrated into the medical training curriculum, and using this model has helped to increase their neurosurgical skills.

### Implications

The training of medical students in Cameroon incorporates the provision of explicit anatomy practicals which includes cadaveric dissections (19). Cadaveric dissections are essential for adequate anatomy exposure amongst African students (7, 20, 21). Despite this knowledge, African countries such as Cameroon are faced with multifaceted barriers to performing dissections including neuroanatomy dissections (10, 11). In this study, almost all the participants had never performed a neuroanatomy/neurosurgery cadaver lab dissection. As a result, these students are forced to turn to other traditional and online resources to acquire anatomy knowledge. In our study, the majority used digital resources for the acquisition of neuroanatomy knowledge. Despite these sources being accessible, they can be misleading, inaccurate (22), and disable the student from the physical experience with cadavers (8, 9). As a result of this learning curve, this will contribute to inadequate neuroanatomy exposure as is the case in this study.

Similar to our study, limited neurosurgery exposure was previously reported in a continental survey by aspiring African neurosurgeons (4). This can be attributed to similar reasons justifying the low exposure to neuroanatomy mentioned above. Also, the lack of an affiliated neurosurgery facility, limited neurosurgery lectures, and ultimately the lack of a neurosurgery mentorship in medical schools are known to be key contributors to this deficit (4). A considerable proportion of participants in this study have neither witnessed or performed a craniotomy, nor have previously used a surgical microscope or neurosurgical instrument. This explains why majority use Textbooks and Youtube to gain neurosurgical experience. Also, the main factors contributing to this deficit include; The paucity of neurosurgical centers in Cameroon limits their exposure (23). Moreover, Cameroon neurosurgeons are overburdened, limiting the time allocated for education and mentorship (24).

Acknowledging the neurosurgical workforce deficit and the burden of neurotrauma in Africa (25, 26), evoked strategies to attain global neurosurgical goals such as task shifting (27, 28) improving undergraduate exposure to neurosurgical practices early in their career is essential (4). Neurosurgery/neuroanatomy exposure can be improved in multiple ways. First and foremost, enhancing mentorship with existing neurosurgery interest groups (29, 30). This will serve as a vehicle to connect medical students, with trainees and consultants in neurosurgery via educational activities. Participating in conferences, symposiums onsite and online has also been advocated (31). Also, the organization by the available neurosurgical centers of continental annual or bi-annual neurosurgery boot camps taking into consideration regional level of excellence and geographical proximity will improve the neurosurgical know-how of undergraduate students (4, 32). A valid strategy to improve at the same time for neurosurgery exposure and cadaver dissection which is crucial to neurosurgical training is the use of hands-on-touch non-cadaver model for neuroanatomy/neurosurgery learning such as the UpSurgeOn models (33, 34).

Gradually migrating from traditional cadaver dissection to cadaver-free laboratories, the place of neurosurgery simulators such as the UpSurgeOn tool is indisputable. Results from our study have it that it is easy to use and easy to get acquainted with, sanctioned by a 60% and 60.9% scores on our study. This is consistent with a similar study carried out using UpSurgeOn tools, where More than 89% of the residents assessed that the application and the augmented reality simulator were very helpful in improving orientation skills during neurosurgical approaches (34). Indeed, 89.3% of participants found brain and skull anatomy highly realistic during their tasks (34). Moreover, workshop exercises were considered useful in increasing the competency and technical skills required in the operating room by 85.8 and 84.7% of residents, respectively (34). The data collected confirmed that the anatomical model and its application were intuitive, well-integrated, and easy to use. However, the lower score in our study (60.9% as opposed to 89%) could be liked to the difference in the academic level of the participants involved, as more of our participants were undergraduates, compared to residents in the latter study, who are considered to have a better appraisal and understanding of the general concepts of neurosurgery, and neuroanatomy.

Given the positive perception of the UpSurgeOn Neurosurgery App, it is not surprising that the majority of our participants would like to use it and advocate for its integration into the national medical curriculum as a teaching tool. This demonstrates its efficiency in facilitating the teaching of neuroanatomy and neurosurgery to imporove knowledge transmission in neurosurgery teaching centers.However, only 24.6% of our participants agreed they would not need the support of a technical person to be able to use UpSurgeOn Box. This emphasizes the degree of independence this tool provides to trainees in the acquisition of neurosurgery and neuroanatomy knowledge.

### Limitations

The results from this study have a few limitations. Our first limitation is that the questionnaire was a one-phase questionnaire. Other studies propose a two-phase questionnaire (pre-test and post-test) to better access the knowledge and practices of participants before and after the training and the impact the training had on their experiences. Our second limitation is that the questionnaire's questions may not have been appropriate to the target population of the survey. Our questionnaire was guided by previous studies, and were filled after the training. The third limitation is that some faculties did not involve the whole student body in the training, as just a few selected classes were allowed to partake in the training due to the measures aimed at reducing overcrowding in line with COVID-19 barrier measures still practiced in Cameroon at the time of our training. The fourth limitation is our sample size which was less than the number of participants in the training. This sample size is also not reflective of the medical student population in Cameroon. We are aware this might affect the generalization of our results. However, the findings gotten are from varied medical schools in Cameroon and truly reflect their realities. Finally, the qualitative nature of this study cannot be undermined, as there was very little quantitative sampling in our study.

### Recommendations

Based on the results of this study, it is appropriate to believe that there is a need for a more inclusive study on this topic involving a majority of students from all medical schools in Cameroon and other African countries. Our results emphasize the impact of simulation tools in facilitating the acquisition of neuroanatomy and neurosurgery knowledge by trainees, therefore stakeholders responsible for policy-making and curriculum drafting for medical schools should consider facilitating the utilization of these simulation tools in the training of our healthcare professionals. Our study provides companies and institutions involved in the production and distribution of simulation tools with evidence of the positive impact of these tools on trainees, this should encourage organizations that are involved in improving surgical education and care in low-resourced settings to provide training institutions in these settings with these simulation tools to accelerate neurosurgery training and increase the neurosurgical workforce in these settings.

## Conclusions

The advent of 3D technologies have evolved and should be exploited to improve all aspects of life, the medical field, and practice. This avenue could be used to install cadaver-free laboratories which are gradually fading out due to ethical and cultural barriers in our context. This will in turn narrow the wide gap that exists between the expected exposure and realities as revealed by the results of this study.

## Data Availability

The original contributions presented in the study are included in the article/**Supplementary Material**, further inquiries can be directed to the corresponding author/s.
