# Supplementary material for: Assessing the impact of neurosurgery and neuroanatomy simulation using 3D non-cadaveric models amongst selected African medical students
Source: Front Med Technol. 2023 May 4;5:1190096. doi: 10.3389/fmedt.2023.1190096 (PMC10192731; doi:10.3389/fmedt.2023.1190096)
Supplement: Supplementary file 1 [file Datasheet1.docx]

**DATA COLLECTION FORM**

**Part 1: Consent**

Have you read, understood the survey information and agree to participate in this study?

1. Yes [ ] B. No [ ]

**Part 2: Basic Sociodemographics**

1. Sex

1. Male [ ] B. Female [ ]

2. Age (in years) …………………

3. Study level

1. Preclinical [ ] B. Clinical [ ]

**Part 3: Previous Experience And Exposure To Neuroanatomy**

1. Neuroanatomy learning tools used prior to the training
2. Atlas = Yes [ ] No [ ]
3. Textbook = Yes [ ] No [ ]
4. Youtube videos = Yes [ ] No [ ]
5. Mobile apps = Yes [ ] No [ ]
6. Cadaver dissection = Yes [ ] No [ ]
7. 3d molded neurostructures = Yes [ ] No [ ]
8. Virtual realities = Yes [ ] No [ ]
9. Dummies = Yes [ ] No [ ]

**Part 4: Previous Experience And Exposure To Neurosurgery**

1. Neurosurgery learning tools used prior to the training
2. Atlas = Yes [ ] No [ ]
3. Textbook = Yes [ ] No [ ]
4. Youtube videos = Yes [ ] No [ ]
5. Mobile apps = Yes [ ] No [ ]
6. Cadaver dissection = Yes [ ] No [ ]
7. 3d molded neurostructures = Yes [ ] No [ ]
8. Virtual realities = Yes [ ] No [ ]
9. Dummies = Yes [ ] No [ ]
10. How many neuroanatomy/neurosurgery cadaver lab dissections have you performed? ....…
11. How will you grade your knowledge of neuroanatomy prior to the training session by UpSurgeon?
12. Excellent [ ] B. Very good [ ] C. Good [ ] D. Fair [ ] E. Poor [ ]
13. How many craniotomies have you witnessed? ………….
14. How many craniotomies have you performed? …………
15. How many craniotomies as FIRST operator have you performed? ……..
16. How many craniotomies as SECOND operator have you performed? …….
17. Type of brainbox used for those who witnessed or performed a craniotomy
18. Pterional = Yes [ ] No [ ] Not applicable [ ]
19. Temporal = Yes [ ] No [ ] Not applicable [ ]
20. Aneurysmal = Yes [ ] No [ ] Not applicable [ ]
21. Retrosigmod = Yes [ ] No [ ] Not applicable [ ]
22. Have you ever used a surgical microscope before? Yes [ ] No [ ]
23. Have you ever used a neurosurgical instruments before? Yes [ ] No [ ]

**Part 5: Previous Experience With The Upsurgeon Neurosurgery Tool**

1. Have you ever used the UpSurgeon Neurosurgery App and the AR simulator? Yes [ ] No [ ]

**Part 6: Perception Of The Upsurgeon Neurosurgery Tool**

1. The BrainBox had an anatomically accurate surface of brain/cerebellum
2. Strongly agree [ ] B. Agree [ ] C. Neutral [ ] D. Strongly disagree [ ] E. Disagree [ ]
3. Neurovascular structures and skull base anatomy were realistic and appropriately detailed for surgical orientation
4. Strongly agree [ ] B. Agree [ ] C. Neutral [ ] D. Strongly disagree [ ] E. Disagree [ ]
5. The tactile feedback to manipulation of the brain/cerebellum was realistic
6. Strongly agree [ ] B. Agree [ ] C. Neutral [ ] D. Strongly disagree [ ] E. Disagree [ ]
7. Feedbacks in the use of a high speed drill and dissection instruments were realistic
8. Strongly agree [ ] B. Agree [ ] C. Neutral [ ] D. Strongly disagree [ ] E. Disagree [ ]
9. The drilling experience was similar to the real skull
10. Strongly agree [ ] B. Agree [ ] C. Neutral [ ] D. Strongly disagree [ ] E. Disagree [ ]
11. Dural opening and suturing was realistic
12. Strongly agree [ ] B. Agree [ ] C. Neutral [ ] D. Strongly disagree [ ] E. Disagree [ ]
13. I found UpSurgeOn Box easy to use
14. Strongly agree [ ] B. Agree [ ] C. Neutral [ ] D. Strongly disagree [ ] E. Disagree [ ]
15. I found the various functions in UpSurgeOn Box were well integrated
16. Strongly agree [ ] B. Agree [ ] C. Neutral [ ] D. Strongly disagree [ ] E. Disagree [ ]
17. I thought there was much consistency in UpSurgeOn Box
18. Strongly agree [ ] B. Agree [ ] C. Neutral [ ] D. Strongly disagree [ ] E. Disagree [ ]
19. I felt very confident using Upsurgeon Box
20. Strongly agree [ ] B. Agree [ ] C. Neutral [ ] D. Strongly disagree [ ] E. Disagree [ ]
21. I needed to learn FEW things before I could get going with UpSurgeOn Box
22. Strongly agree [ ] B. Agree [ ] C. Neutral [ ] D. Strongly disagree [ ] E. Disagree [ ]

**Part 7: Attitudes Towards The Upsurgeon Neurosurgery Tool**

1. This method of training should be part of a standard training curriculum, in addition to traditional training methods
2. Strongly agree [ ] B. Agree [ ] C. Neutral [ ] D. Strongly disagree [ ] E. Disagree [ ]
3. I think that I would like to use UpSurgeOn Box frequently
4. Strongly agree [ ] B. Agree [ ] C. Neutral [ ] D. Strongly disagree [ ] E. Disagree [ ]
5. I think that I would NOT need the support of a technical person to be able to use UpSurgeOn Box
6. Strongly agree [ ] B. Agree [ ] C. Neutral [ ] D. Strongly disagree [ ] E. Disagree [ ]
7. I would imagine that most people would learn to use UpSurgeOn Box very quickly

A.Strongly agree [ ] B. Agree [ ] C. Neutral [ ] D. Strongly disagree [ ] E. Disagree [ ]

**Part 8: Impact Of The Upsurgeon Neurosurgery Tool To Neurosurgical Training**

1. The Neurosurgery App and the AR simulator help to develop the orientation skills needed during neurosurgical approach, in addition to traditional resources.

A.Strongly agree [ ] B. Agree [ ] C. Neutral [ ] D. Strongly disagree [ ] E. Disagree [ ]

1. Using this model helps to increase familiarity and to acquire neurosurgical skills

A.Strongly agree [ ] B. Agree [ ] C. Neutral [ ] D. Strongly disagree [ ] E. Disagree [ ]

1. After using this model I feel more confident in the use of neurosurgical instruments

A.Strongly agree [ ] B. Agree [ ] C. Neutral [ ] D. Strongly disagree [ ] E. Disagree [ ]

1. After using this model I feel more confident in the use of the surgical microscope

A.Strongly agree [ ] B. Agree [ ] C. Neutral [ ] D. Strongly disagree [ ] E. Disagree [ ]

1. The sequence of mental training (app), hybrid training (Augmented Reality) and manual training (BrainBox) is an effective method of training in order to fill the gap between theoretical knowledge and practice on a real patient/cadaver

A.Strongly agree [ ] B. Agree [ ] C. Neutral [ ] D. Strongly disagree [ ] E. Disagree [ ]
